# Differentiating Individuals with and without Alcohol Use Disorder Using Resting-State fMRI Functional Connectivity of Reward Network, Neuropsychological Performance, and Impulsivity Measures

**DOI:** 10.3390/bs12050128

**Published:** 2022-04-28

**Authors:** Chella Kamarajan, Babak A. Ardekani, Ashwini K. Pandey, Sivan Kinreich, Gayathri Pandey, David B. Chorlian, Jacquelyn L. Meyers, Jian Zhang, Elaine Bermudez, Weipeng Kuang, Arthur T. Stimus, Bernice Porjesz

**Affiliations:** 1 Henri Begleiter Neurodynamics Lab, Department of Psychiatry and Behavioral Sciences, SUNY Downstate Health Sciences University, Brooklyn, NY 11203, USA; ashwini.pandey@downstate.edu (A.K.P.); sivan.kinreich@downstate.edu (S.K.); gayathri.pandey@downstate.edu (G.P.); david.chorlian@downstate.edu (D.B.C.); jacquelyn.meyers@downstate.edu (J.L.M.); jian.zhang@downstate.edu (J.Z.); weipeng.kuang@downstate.edu (W.K.); arthur.stimus@downstate.edu (A.T.S.); bernice.porjesz@downstate.edu (B.P.); 2 Center for Advanced Brain Imaging, Nathan Kline Institute for Psychiatric Research, Orangeburg, NY 10962, USA; babak.ardekani@nki.rfmh.org; 3 Department of Psychiatry, NYU School of Medicine, New York, NY 10016, USA; bermue01@nyu.edu

**Keywords:** alcohol use disorder (AUD), random forests (RF), resting-state functional connectivity (FC), reward network (RN), functional MRI (fMRI), neuropsychological tests, impulsivity

## Abstract

Individuals with alcohol use disorder (AUD) may manifest an array of neural and behavioral abnormalities, including altered brain networks, impaired neurocognitive functioning, and heightened impulsivity. Using multidomain measures, the current study aimed to identify specific features that can differentiate individuals with AUD from healthy controls (CTL), utilizing a random forests (RF) classification model. Features included fMRI-based resting-state functional connectivity (rsFC) across the reward network, neuropsychological task performance, and behavioral impulsivity scores, collected from thirty abstinent adult males with prior history of AUD and thirty CTL individuals without a history of AUD. It was found that the RF model achieved a classification accuracy of 86.67% (AUC = 93%) and identified key features of FC and impulsivity that significantly contributed to classifying AUD from CTL individuals. Impulsivity scores were the topmost predictors, followed by twelve rsFC features involving seventeen key reward regions in the brain, such as the ventral tegmental area, nucleus accumbens, anterior insula, anterior cingulate cortex, and other cortical and subcortical structures. Individuals with AUD manifested significant differences in impulsivity and alterations in functional connectivity relative to controls. Specifically, AUD showed heightened impulsivity and hypoconnectivity in nine connections across 13 regions and hyperconnectivity in three connections involving six regions. Relative to controls, visuo-spatial short-term working memory was also found to be impaired in AUD. In conclusion, specific multidomain features of brain connectivity, impulsivity, and neuropsychological performance can be used in a machine learning framework to effectively classify AUD individuals from healthy controls.

## 1. Introduction

The drugs of abuse, including alcohol, exert and maintain their reinforcing effects through the reward circuitry of the brain [1,2]. Neuroimaging studies have documented the disruption of reward processing in addiction [3] and implicated brain reward circuitry in different stages of alcohol use disorder (AUD) [4]. The reward network, in the context of addiction, primarily includes structures from the midbrain (i.e., ventral tegmental area, VTA), basal ganglia (i.e., nucleus accumbens, NAc; caudate nucleus; and putamen), limbic system (i.e., amygdala, hippocampus, and thalamus), and cerebral cortical regions (i.e., prefrontal cortices, insula, cingular cortices, and superior and inferior parietal lobules) [1,2,5,6,7]. Deficient reward processing due to abnormalities in the reward network in individuals with chronic AUD has been widely reported for both monetary and drug-related rewards [8,9,10,11,12,13,14,15,16]. For instance, an fMRI finding reported by Wrase et al. [10] showed that the ventral striatum (VS), a key reward region, showed increased activation for alcohol-related cues, but decreased activation for monetary gain in detoxified alcoholics, relative to healthy controls, suggesting differential activation of VS in response to the type of reward. Both animal and human studies have revealed that chronic administration of addictive substances results in neuroadaptations in reward structures, especially in the midbrain dopaminergic areas (e.g., VTA, substantia nigra), as well as the structures to which they project (e.g., NAc) [17,18].

Nevertheless, brain network interactions between mesocorticolimbic regions and other subcortical and cortical structures, especially in human participants with chronic AUD, have not been well-understood [19]. Historically, dysregulation in brain dopaminergic system has been postulated as the primary mechanism underlying substance use disorders (SUD) [20,21], and, therefore, studies have attempted to elucidate alterations in the reward network in human patients with SUD (cf. Sutherland et al. [19]). The integrity and communication strength of neural networks have been measured using functional connectivity [22], which is defined as the temporal dependency of neuronal activation patterns of anatomically separated brain regions [23]. Resting-state functional connectivity (rsFC) provides a reliable approach for studying various networks underlying ongoing cognitive and emotional processes [24]. Since the phenomenon was first reported by Biswal et al. [25], a growing number of studies have examined rsFC and elucidated brain functioning, in terms of neural communication in both healthy subjects and patients with various neurological and psychiatric [24,26,27] and substance use disorders (SUD) [28,29], including AUD [30,31,32,33,34,35,36]. However, to our knowledge, there have been no studies exploring both cortical and subcortical reward networks during resting state in abstinent individuals with past history of AUD, and who manifest a variety of neurocognitive deficits [37]. Since studying detoxified and abstinent AUD individuals relative to healthy controls without past AUD has an advantage in determining the brain impacts due to chronic past drinking without the confounding effects of current drinking [8,38,39], the current study provides an important opportunity to investigate the resting-state functional connectivity of the reward network in abstinent individuals with past AUD.

Studies have also shown that abstinent individuals with AUD often manifest poor neuropsychological performance [40,41,42,43,44,45] and heightened impulsivity [46,47,48], and, therefore, it is important to include these domains in the models exploring concomitants and/or determinants of alcohol-related outcomes. In our previous study, we reported that a random forest algorithm was highly useful for classifying AUD individuals from controls using multi-modal measures, including fMRI functional connectivity of the default mode network, neuropsychological performance, and impulsivity. Since AUD and other addiction traits are shown to be associated with reward networks [49,50] and as substance induced neuroadaptation primarily involves reward structures [51], the current study aimed to examine the functional connectivity across the reward network, along with measures of neuropsychological performance and impulsivity, to differentiate abstinent AUD individuals from healthy controls. Similar to our previous study, we computed the predictive power of these multi-domain measures in terms of classification accuracy in a machine learning framework and evaluated the utility of these phenotypic features, especially the reward network connectivity measures. Since recent studies have proposed that resting state fMRI connectivity can potentially serve as one of the key neuroimaging biomarkers for quantitative clinical evaluation of AUD [36,52,53], the findings from the current study may elucidate specific connectivity patterns across reward regions of the brain in abstinent AUD individuals, along with key neuropsychological and impulsivity features, which are distinctly different from healthy controls.

## 2. Materials and Methods

**Figure 1:**
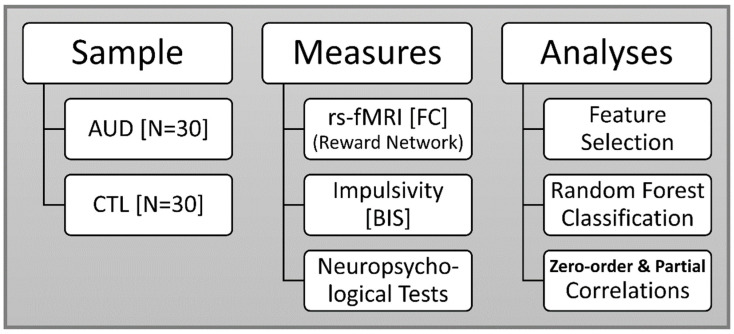
The study protocol listing the sample, measures, and analytic techniques. The sample consisted of two groups of 30 individuals each, viz., AUD and CTL. The measures used in the prediction model included rs-fMRI functional connectivity (reward network), impulsivity assessed with Barratt impulsiveness scale (BIS), and neuropsychological performance scores. Major analyses were features selection for selecting FC variables, random forest classification method, and correlational analyses, including zero-order and partial correlations.

The study protocol is illustrated in Figure 1. The sample consisted of 30 abstinent individuals with past diagnosis of AUD and 30 healthy volunteers (CTL) (see Section 2.1 for details). The analytic measures included rs-fMRI (Section 2.4, Section 2.5 and Section 2.6), self-rated impulsivity scores (Section 2.3), and neuropsychological test scores (Section 2.2). Major analyses employed in the study were (i) feature selection to extract relevant features that will be used in classification analysis (Section 2.7), (ii) random forest method to identify the key features that significantly contribute to classifying AUD from the CTL participants (Section 2.8), (iii) zero-order correlations were used to compute associations of the key variables identified by the random forest classification model, (a) among themselves (Section 3.2) and (b) with age in each group (Section 3.3). Partial correlations were employed to identify associations between age and the key variables of classification by controlling the group effects in the total sample (Section 3.3).

### 2.1. Sample

The sample characteristics are presented in Table 1, and a detailed description is also available in Pandey et al. [54]. All participants in the current study were drawn from the sample of a larger study on brain dysfunction in chronic alcoholism conducted at the SUNY Downstate Health Sciences University, Brooklyn, NY, USA. Thirty currently abstinent adult males with past AUD (mean age (SD) = 41.42 (7.31) years) and thirty unaffected male controls (mean age (SD) = 27.44 (4.74) years), who had undergone multimodal assessments, including structural and functional MRI and neuropsychological tests, were selected for the present study. The “race/ethnic” distribution of the sample was: African Ancestry = 25; European Ancestry = 9; Asian = 21; American Indian = 1; More than one race = 2; and Unknown = 2. Participants with AUD were recruited from alcohol treatment centers in and around New York City after they had been detoxified and abstinent for at least 30 days prior to testing. As shown in Table 1, some of the participants from the AUD group had consumed tobacco (N = 20) and/or marijuana (N = 10) during the last 6 months (but not at least 5 days before testing). None of the participants were in withdrawal for alcohol or any other drugs (including nicotine) at the time of testing. Individuals for the control group (CTL) were recruited through advertisements and screened to exclude any personal or family history of major medical, psychiatric, or substance-related disorders. The CTL participants did not have any past or present history of substance dependence or abuse (DSM-IV), although some of them (N = 12) were light/regular drinkers and had used alcohol in the last 6 months (N = 18) (see Table 1 for details). All participants were asked to abstain from alcohol and other drugs for 5 days prior to MRI scans. Clinical information regarding substance use, psychiatric disorder, and family history were assessed using a modified version of the semi-structured assessment of genetics of alcoholism (SSAGA) [55]. The majority of subjects were right-handed, with only a few who were either left-handed (5 in AUD and 2 in CTL group) or bi-dexterous (2 in AUD and 1 in CTL group). Clinical and psychometric data were collected at the SUNY Downstate Health Sciences University, while the fMRI data were acquired at the Nathan Kline Institute (NKI) for Psychiatric Research. Standard MRI safety protocols and exclusion criteria (implants, tattoos, cosmetics, claustrophobia, etc.) were followed to ensure subjects’ safety and data quality. Individuals with hearing/visual impairment, a history of head injury, or moderate and severe cognitive deficits (<21) on mini-mental state examination (MMSE) [56] were also excluded from the study. All participants provided informed consent and the Institutional Review Boards of both centers approved the research protocols (IRB approval ID: SUNY–266893; NKI–212263).

### 2.2. Neuropsychological Assessment

Participants were administered two computerized tests from the Colorado assessment tests for cognitive and neuropsychological assessment [57], namely, the Tower of London Test (TOLT) [58], and the visual span test (VST) [59,60].

#### 2.2.1. Tower of London Test (TOLT)

The TOLT assesses planning and problem-solving ability of the executive functions. In this test, participants were asked to solve a set of puzzles with graded difficulty levels by arranging a number of colored beads one at a time from a starting position to the desired goal position in as few moves as possible. The test consisted of 3 puzzle types, with 3, 4, and 5 colored beads placed on the same number of pegs, with 7 problems/trials per type and a total of 21 trials. The following performance measures from the sum total of all puzzle types were used in the analysis: (i) excess moves, which is the additional moves beyond the minimum moves required to solve the puzzle (“ExcMovMade_All”); (ii) average pickup time, which is the initial thinking/planning time spent until picking up the first bead to solve the puzzle (“AvgPicTime_All”); (iii) average total time, which is the total thinking/planning time to solve the problem in each puzzle type (“AvgTotTime_All”); (iv) total trial time, which is the total performance/execution time spent on all trials within each puzzle type (“TotTrlTime_All”); and (v) average trial time, which is the mean performance/execution time across trials per puzzle type (“AvgTrlTime_All”).

#### 2.2.2. Visual Span Test (VST)

The VST measured visuospatial memory span from the forward condition and working memory from the backward condition. In this test, a set of randomly arranged squares, ranging from 2 to 8 squares per trial, flashed in a predetermined sequence depending on the span level being assessed. Each span level was administered twice, with a total of 14 trials in each condition. During the forward condition, subjects were required to repeat the sequence in the same order by clicking on the squares using a computer mouse. In the backward condition, subjects were required to repeat the sequence in the reverse order (starting from the last square). The following performance measures were collected during forward and backward conditions: (i) total number of correctly performed trials (“TotCor_Fw” and “TotCor_Bw”); (ii) maximum span or sequence-length achieved (“Span_Fw” and “Span_Bw”); (iii) total average time, which is the sum of mean time-taken across all trials performed (“TotAvgTime_Fw” and “TotAvgTime_Bw”); and (iv) total correct average time, which is the sum of mean time-taken across all trials correctly performed (“TotCorAvgTime_Fw” and “TotCorAvgTime_Bw”).

### 2.3. Impulsivity Scores

Barratt Impulsiveness Scale—Version 11 (BIS-11) [61], a 30-item self-administered tool with excellent psychometric properties [62], was used to assess aspects of impulsivity. All three subscales, viz., motor impulsivity (BIS_MI), non-planning (BIS_NP), and attentional impulsivity (BIS_AI), were included in the random forest model.

### 2.4. MRI Data Acquisition

The details of fMRI data acquisition on the same sample have been described previously by our group [63]. Briefly, MRI scanning was performed at the Nathan Kline Institute using a 3.0 Tesla Siemens Tim Trio scanner (Erlangen, Germany). The resting-state fMRI data, used in the current study, were collected during eyes closed alert state for 6.11 s. A series of T2*-weighted gradient echo single-shot *echo-planar imaging* (EPI) volumes with the following sequence parameters was acquired: TR = 2750 ms; TE = 30 ms; flip angle = 80°; voxel size = 2.5 × 2.5 × 3.5 mm^3^; matrix size = 96 × 96; number of slices = 50; number of volumes = 130; FOV = 240 mm; and Grappa acceleration factor = 3. The sequence was carefully optimized to minimize the effects of magnetic susceptibility inhomogeneities (such as distortions and signal dropouts), as well as the effects of mechanical vibrations, which elevate Nyquist ghosting levels. In addition, *a magnetization-prepared rapid gradient-echo* (MPRAGE) high-resolution three-dimensional T1-weighted structural image served as an anatomical reference for the fMRI data, as well as for the non-linear registration of imaging data between subjects. The sequence parameters for the MPRAGE were: TR = 2500 ms; TE = 3.5 ms; TI = 1200 ms; flip angle = 8°; voxel size = 1 × 1 × 1 mm^3^; matrix size = 256 × 256 × 192; FOV = 256 mm; and number of averages = 1.

### 2.5. Image Processing

The details of image processing for the resting state fMRI are available in our previous study [63]. Briefly, the intra-subject inter-modality linear registration module [64] of the automatic registration toolbox (ART; www.nitrc.org/projects/art (accessed on 20 December 2019) was used to register the structural (MPRAGE) and functional MRI volumes. The *brainwash* program within the ART toolbox was used for skull-stripping the MPRAGE volumes. Motion detection and correction were performed using the *3dvolreg* module of the AFNI software package [65]. Furthermore, the non-linear registration module of the ART [66] was used to correct for the geometric distortions of the fMRI images due to magnetic susceptibility differences in the head, particularly at brain/air interfaces. The skull-stripped MPRAGE images from all subjects were non-linearly registered to a study-specific population template using ART’s non-linear registration algorithm, which is one of the most accurate inter-subject registration methods available [67]. The population template was formed using an iterative method [68], and the motion-corrected fMRI time-series were detrended using PCA [69]. Finally, fMRI images from all subjects were normalized to a standard space.

### 2.6. Reward Network Seed Regions and rsFC Calculations

**Figure 2:**
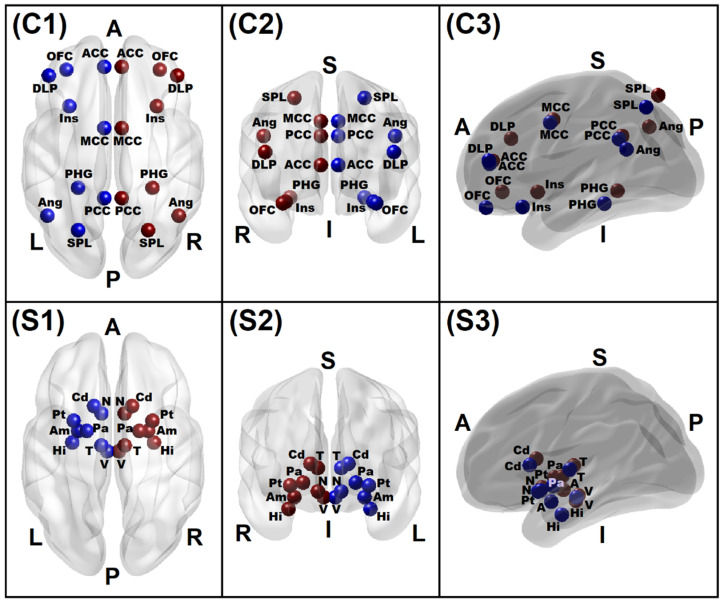
Brain reward network regions of interest comprising 16 cortical areas (top panels, **C1**–**C3**) and 18 subcortical areas (bottom panels, **S1**–**S3**), as listed in Table 1. *Bilateral cortical regions:* Insula (Ins), Parahippocampal Gyrus (PHG), Anterior Cingulate Cortex (ACC), Mid-Cingulate Cortex (MCC), Mid-Cingulate Cortex (MCC), Posterior Cingulate Cortex (PCC), Orbitofrontal Cortex (OFC), Angular Gyrus (Ang), Superior Parietal Lobule (SPL), Dorsolateral (PFC), and Dorsolateral Prefrontal Cortex (DLP). *Bilateral subcortical regions:* Ventral Tegmental Area (V), Nucleus Accumbens (N), Amygdala (A), Hippocampus (Hi), Caudate (Cd), Pallidum (Pa), Thalamus (T), and Putamen (Pt). *Colors:* Blue—Left hemispheric regions; Red—Right hemispheric regions. *Views:* C1/S1—Axial top view; C2/S2—Coronal front view; C3/S3—Sagittal left view. *Directions:* A—Anterior; P—Posterior; S—Superior; I—Inferior; L—Left; R—Right.

The regions of interest (ROIs) for the reward network were identified from the published literature of review and meta-analyses of reward processing, e.g., [6,70,71]. These included 34 ROIs from 17 bilateral structures involving nine bilateral subcortical structures and eight bilateral cortical regions (Table 2 and Figure 2). The ROIs were marked using ITK-SNAP, an image processing application [72]. The diameters of subcortical and cortical ROIs were 7 mm and 11 mm, respectively, from the MNI coordinates (Table 2). The ROI-to-ROI connectivity [73], the most commonly used method to derive rsFC across brain regions [74], was computed using Pearson correlation coefficients between all unique pairs (N = 561) of BOLD time series data of all 34 ROIs listed in Table 2. The resulting correlation coefficients were Fisher Z-transformed for further statistical analyses. All 561 connections derived from unique combinations of the ROIs were used in the feature selection process (see Section 2.7), which provided the subset of features to be used in the random forest model (see Section 2.8).

### 2.7. Feature Selection of FC Variables

Recent approaches dealing with machine learning analyses have used a two-stage approach, consisting of feature selection followed by a predictive algorithm using a selected sets of variables [75,76,77,78,79]. Feature selection methods are used as the first stage to reduce irrelevant and redundant variables, which may otherwise add noise to the predictive models [75,76,77]. The advantages of feature selection include a better understanding of the data, reducing computation requirements, mitigating the effect of the curse of dimensionality, and also improving the predictor performance [76]. We applied binomial lasso regression [80,81,82] as a feature selection method [83,84], as implemented in R-package “glmnet”, to extract a subset of fMRI FC variables (N = 561) that held significant predictive value to discriminate AUD from the CTL group. Feature selection was implemented only for the rsFC variables, due to a high number of connections, most of which were deemed not relevant for our purpose of AUD classification. The method adapted in the current analysis is based on the Lasso method, as implemented in Fonti and Belitser [84]. The maximum number of output features “pmax” was set to 10% (i.e., 56 of the total 561 variables). A 10-fold cross-validation and lambda thresholding with 1 SE (λ_1se_) were set to extract the final set of key variables. The area under the curve (AUC) was plotted to determine the classification performance of the selected features. The final subset resulting from the feature selection process included 21 rsFC variables (see Table A1, Appendix A).

### 2.8. Random Forest Classification

The random forest classification model, as used in the current study, has been described in our previous work on rsFC of the default mode network (DMN) [63]. The predictor variables included in the model were 21 reward network connections identified by feature selection (Table A1), 13 neuropsychological scores consisting of 5 TOLT scores and 8 VST scores (see Section 2.2), and 3 BIS scores (see Section 2.3), while the group status (AUD and CTL) served as the outcome variable (see Table A1, Appendix A). Although age was significantly different between the groups, we did not include age as an input variable in the classification model for the following reasons: (i) as done in our previous publications on the same sample of subjects [63,85], we performed post hoc correlational analysis of age with the significant features of the random forest model, to see if any of the top variables had associations with age in the individual groups or in total sample (see Section 3.4); and (ii) since the age difference between the groups was highly significant, including age as a feature in the classification model would likely artificially increase the accuracy of classification, which would not be desirable. To compute the classification accuracy of the random forest model, we used an out-of-bag (OOB) error estimate. According to Breiman and Cutler [86], in random forests, owing to the inbuilt OOB feature in the model, additional cross-validation is not a requirement to obtain an unbiased estimate of the test sample error, since it is estimated internally in the OOB algorithm. The random forest algorithm constructs each of the decision trees using separate bootstrap subsamples from the training data, and about one-third of the observations from the training data are left out during each bootstrap, called the OOB sample, which are used as a form of test data only, to estimate the prediction accuracy of the RF model. While classification trees are grown for each bootstrap sample (which is approximately two-thirds of the training data), the OOB error rate is calculated for each classification tree built. According to Breiman [87], there are two reasons for using bagging: (i) to enhance the accuracy when random features are used; and (ii) to give ongoing estimates of the generalization error of the combined ensemble of trees, as well as estimates for the strength and correlation. The aggregate of OOB scores on all “ntree” trees (which is the maximum number of trees pre-set in the model calculation) provides the ensemble OOB error rate. Thus, the OOB score provides validation for the RF model. Therefore, unlike in other machine learning algorithms, random forests method does not require separate training data and test data while specifying the model term. In the current study, the maximum number of trees “ntree” was set at 1000. The optimal number of features analyzed at each node (“Mtry”) in the model was estimated to be 8 (using the “tuneRF” function). The final list of variables that significantly contributed to the classification was tabulated and sorted based on their importance to classification. For the top significant FC variables, the brain connectivity across ROIs was mapped onto a 3-dimensional ICBM atlas [88] using custom Matlab scripts.

## 3. Results

### 3.1. Random Forests Classification

#### 3.1.1. Classification Accuracy and Top Significant Variables

**Figure 3:**
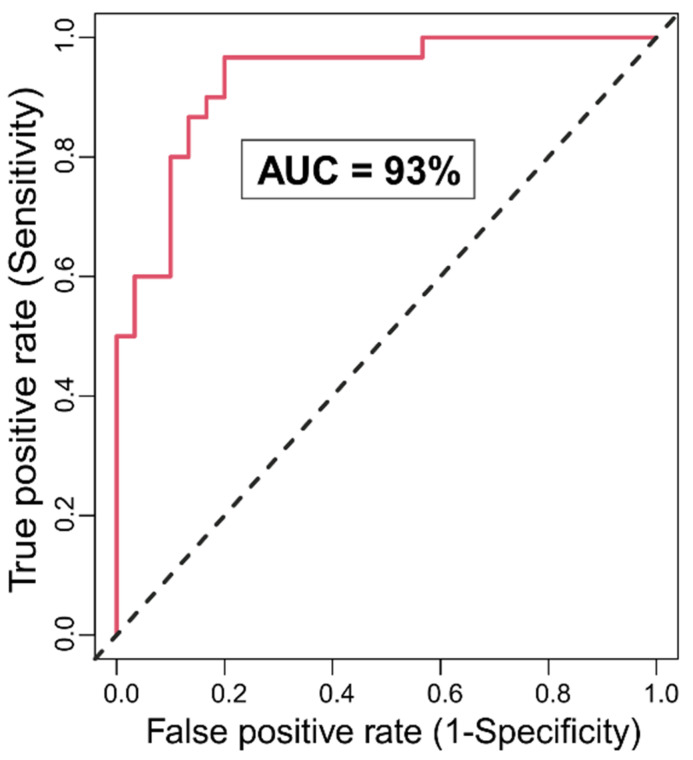
Receiver operating characteristic (ROC) curve for the RF model for different true positive rate (sensitivity) and false positive rate (1-specificity) are shown. The area under the ROC curve was 93%. (The further away the curve from the diagonal dashed line and the closer the swelling to the top left corner, the better the classification).

The RF algorithm correctly identified group membership of 26 out of 30 individuals in each group, in classifying them into either AUD or CTL group, with an accuracy rate of 86.67% and the area under the curve of 93% (Figure 3). The OOB error or the misclassification rate was 13.33% (for each group). The model also identified 12 rsFC connections and two impulsivity scores (motor and non-planning) as significantly (*p* < 0.05) contributing to the classification (Table 3). Relative to the CTL individuals, AUD subjects showed a predominant pattern of hypoconnectivity (i.e., decreased rsFC in 9 out of 12 connections) across the major cortical and subcortical nodes of the reward network, in addition to three connections with hyperconnectivity in specific nodes (i.e., left nucleus accumbens–left posterior cingulate cortex (PCC), right pallidum–right PCC, and right hippocampus–left dorsolateral prefrontal cortex). AUD individuals also showed increased impulsivity in motor and non-planning categories. However, none of the neuropsychological variables were significant based on the *p*-value criterion.

#### 3.1.2. Distribution of Minimal Depth

**Figure 4:**
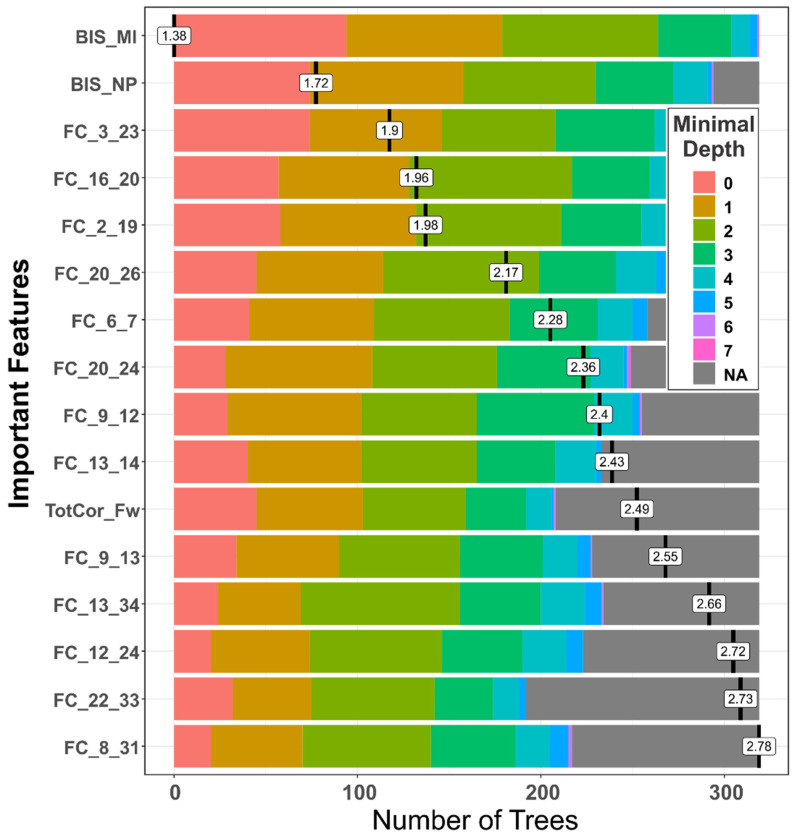
The distribution of minimal depth among the trees of the forest for the significant variables is shown in different colors for each level of minimal depth. The mean minimal depth in the distribution for each variable is marked by a vertical black bar overlapped by a value label inside a box. Based on the *mean minimal depth* values, the importance list comprised 2 BIS scores, 13 FC, and 1 neuropsychological score, which contributed to the RF classification of AUD and CTL individuals. The lower mean minimal depth of a feature represents a higher number of observations (participants) categorized in a specific group based on the feature. The number of trees for a feature represents the total number of decision trees in which a split occurs on the feature (see Table 2 for details about the ROI numbers (1–34) represented in the FC variables).

The distribution of minimal depth among the decision trees of the forests for the top significant variables is shown in Figure 4. Minimal depth of a variable represents the depth of the node that splits on that variable and is the closest to the root of the decision tree. The lower mean minimal depth of a variable represents a higher number of observations (participants) categorized in a specific group based on that variable. The ranking based on the *minimal depth* parameter shows that two of the impulsivity variables are a the top of the importance list, followed by several reward network connections and a neuropsychological feature (total correct score in the forward trials of the visual span test).

#### 3.1.3. Multi-Way Importance

**Figure 5:**
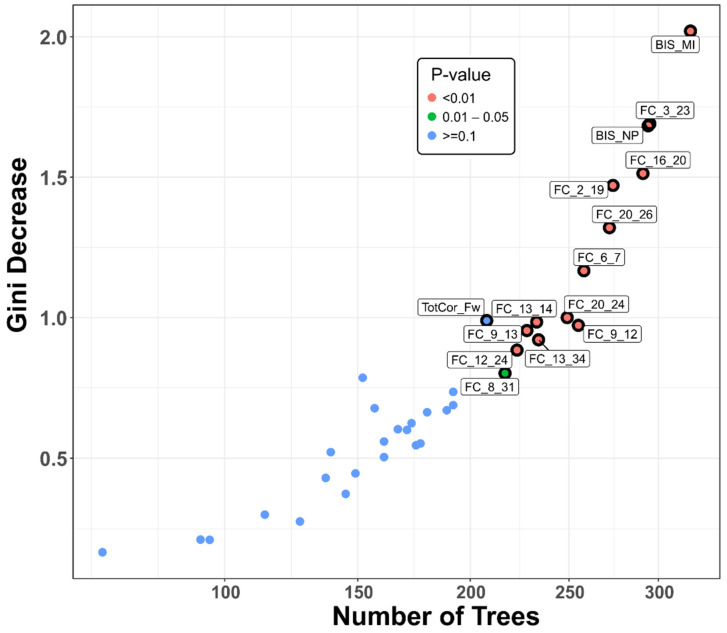
Multi-way importance plot showing the top significant features (labeled and marked with black circles) that contributed to the classification of alcohol use disorder from control individuals based on the measures *Gini decrease* (mean decrease in node impurity or classification error), *number of trees* (total number of decision trees in which a split occurred), and *p-value* (probability of node splits). The top variables of importance included 2 impulsivity scores, 12 rsFC connections, and 1 neuropsychological variable (see the circled and labeled dots). Notations in the variable labels: BIS–Barratt Impulsivity Scale; MI–Motor impulsivity; NP–Non-planning; TotCor_Fw–Total correct forward. ROIs of FC variables: Refer to Table 2 for details about the ROI numbers (1-34) that are represented in the rsFC variable names.

The top significant variables can also be shown by a multi-way importance plot based on any of the three RF importance measures. Figure 5 illustrates the important features that contributed to group classification based on *Gini decrease*, *number of trees*, and *p-value*. These features include: (i) 12 FC variables (representing connections across cortical and subcortical regions) (see Table 2 for details), (ii) one neuropsychological variable (i.e., total number of correct trials in forward span), and (iii) two impulsivity scores (motor and non-planning categories).

#### 3.1.4. Correlations among Rankings of RF Parameters

**Figure 6:**
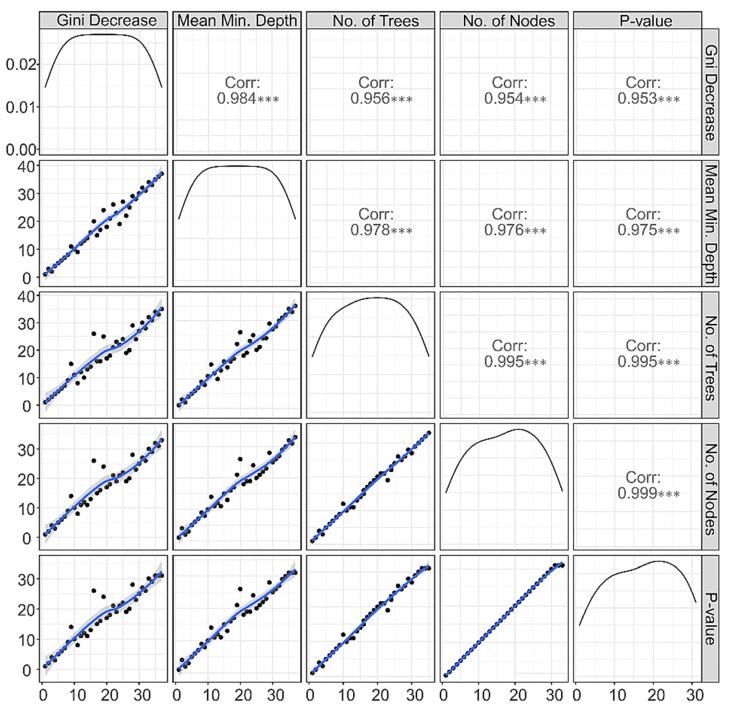
Illustration of rankings of features based on correlation between any two random forest (RF) parameters. The panels in the lower triangle of the grid show the distribution of rankings of all predictor variables with black dots along a blue trend line. The panels in the upper triangle of the grid show correlation coefficients across rankings of any two parameters. It is shown that all RF parameters of importance were found to have very high correlations among each other, suggesting the high reliability of each of these parameters in ranking the importance of features for classification. The asterisks (***) represents that the correlations were highly significant (*p* < 0.001).

The correlations among the rankings of features based on different RF parameters (Figure 6) were very high and significant (r > 0.9), suggesting that each of the RF parameters would rank the features in a very similar order while classifying the individuals into either the AUD or CTL group. High correlations among these parameters also suggest that each parameter is very valuable and reliable in terms of its classification performance, lending further support to the utility of the RF technique as a powerful tool for classifying individuals using a set of multi-domain features.

#### 3.1.5. Connectivity Mapping of Significant rsFC Connections

**Figure 7:**
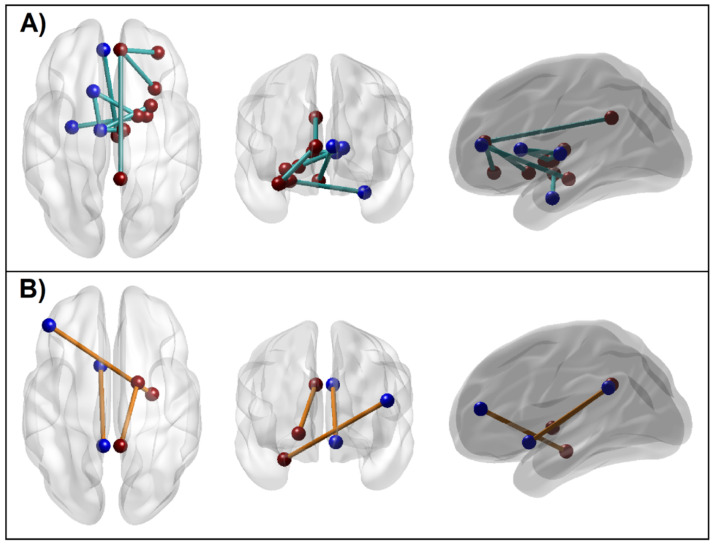
Significant reward network connections that contribute to the random forest classification of alcohol use disorder (AUD) from control (CTL), as listed in Table 3. (**A**): Hypoconnectivity manifested by the AUD group (cyan lines) across cortical and subcortical ROI regions of the RN, predominantly involving right hemisphere structures; (**B**): Hyperconnectivity in three connections manifested by the AUD group (orange lines). Images within each panel: *Left:* axial (top) view; *Middle:* coronal (front) view; *Right:* sagittal (left) view.

Significant reward network connections are illustrated in Figure 7. Among the 12 significant connections, nine were hypoconnected and three were hyperconnected in AUD individuals, involving 17 regions (7 from the left and 10 from the right hemisphere) of the 34 reward network ROIs. While the majority of these nodes (12 of 17) were of solo paths, connecting to another single node (ROI # 2, 3, 6, 7, 8, 14, 16, 19, 23, 26, 31, 34), two of them (ROI # 13, 20) were linked with three connections each, and three of them (ROI # 9, 12, 24) had two connections each. Out of nine hypoconnected FC variables, three were cortico-cortical connections (R.Ins–R.ACC, R.ACC–R.OFC, and R.ACC–R.PCC), involving only the right hemisphere, and five were subcortical–subcortical connections (R.Amg–L.Hip, L.Cdt–R.Pal, L.Tha–R.Tha, L.Cdt–L.Tha, and L.Tha–R.Ptm) involving both hemispheres, and one inter-hemispheric subcortical–cortical connection (R.VTA–L.ACC). Interestingly, all three hyperconnected FC variables were subcortical–cortical connections, involving a left-hemispheric connection (L.NAc–L.PCC), a right-hemispheric connection (R.Pal–R.PCC), and an inter-hemispheric connection (R.Hip–L.DLP).

### 3.2. Correlations among the Top Significant Variables

**Figure 8:**
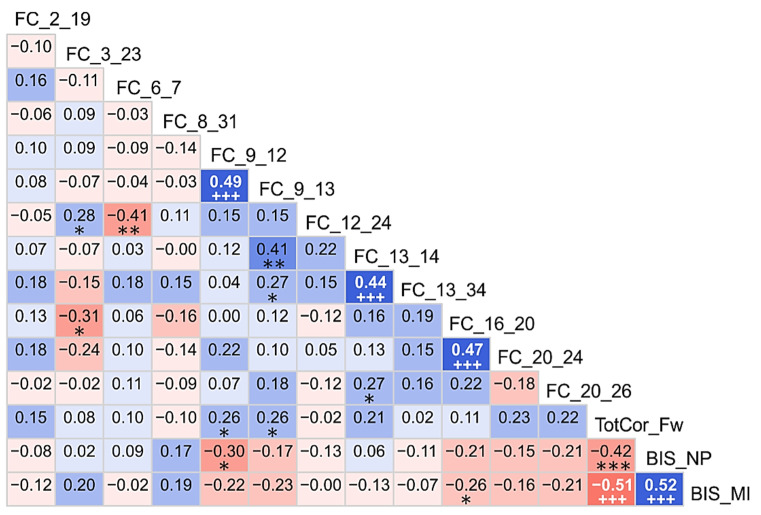
Correlation matrix showing associations among the top significant variables based on explorative (descriptive) correlational analysis for the interpretative purpose. Values within each cell represent a bivariate Pearson correlation between the variable on its vertical axis and the variable on its horizontal axis. Correlation coefficients are color-coded (red/pink shades represent negative r-values, blue/cyan shades indicate positive r-values, darker color represent higher magnitude) and significant correlations (before Bonferroni correction) have been marked with asterisks in black font (* *p* < 0.05; ** *p* < 0.01; *** *p* < 0.001). The significant correlations that survived Bonferroni correction have been marked with +++ sign in white font (+++ *Significant after Bonferroni correction*). Refer to Table 2 for details about the ROI numbers (1–34) that are represented in the rsFC variable names. *Acronyms*: FC–Functional connectivity, TotCor_Fw–total number of correctly performed forward trials, BIS–Barratt Impulsiveness Scale, NP–Non-planning, MI–Motor Impulsivity.

Exploratory (descriptive) analysis using zero-order correlations among the top significant variables is shown in Figure 8. Only five positive correlations survived Bonferroni correction for multiple comparisons (as represented by the sign +++), which include: (i) three positive correlations between FC features (L.Cdt–R.Pal with L.Cdt–L.Tha; L.Tha–R.Tha with L.Tha–R.Ptm; and R.Ins–R.ACC with R.ACC–R.PCC), (ii) a negative correlation between memory recall and motor impulsivity, and (iii) a positive correlation between non-planning and motor impulsivity. Each of the significant FC correlations had a common node, i.e., L.Cdt, L.Tha, and R.ACC, respectively.

### 3.3. Correlations between Significant Variables and Age

Since the age difference across the groups was statistically significant (*p* < 0.001), the association of age with significant predictor variables from the RF analysis was calculated within each group using bivariate Pearson correlation and in the total sample using partial correlation adjusted for the group effect (Table 4) as an exploratory (descriptive) analysis. It was found that there was no significant association of age with the top significant variables in any of the groups or in the total sample, after correcting for multiple comparisons. However, a single FC variable (FC_6_7) representing the connectivity between the right amygdala and left hippocampus was significant (r = 0.38; *p* = 0.0374), but it did not survive multiple testing correction.

### 3.4. Neuropsychological Scores between the Groups

Since the rankings of neuropsychological variables varied widely across different parameters of the random forest classification, these features were statistically compared across the participant groups using one-way analysis of variance (ANOVA) to determine the level of significance (see Table 5). Only two variables from the visual span test (i.e., TotCor_Fw and Span_Fw) were significant after Bonferroni corrections. The score “TotCor_Fw” (total number of correctly performed forward trials), which showed the highest significance level, was also identified by some of the parameters of the random forest as a variable contributing to group classification.

## 4. Discussion

The goal of the present study was to identify specific features from a set of multi-domain measures, including functional connectivity in the reward network, neuropsychological performance, and impulsivity, to classify individuals with AUD from healthy controls. The results showed that the random forest algorithm was highly successful in identifying the key features that contributed significantly to differentiating AUD from CTL individuals. Relative to controls, AUD individuals manifested (i) alterations in functional connectivity across reward network regions (including ventral tegmental area, nucleus accumbens, anterior insula, anterior cingulate cortex, and other cortical and subcortical structures), showing hypoconnectivity in nine connections and hyperconnectivity in three connections, (ii) increased impulsivity in motor and non-planning categories, and (iii) poorer neuropsychological performance, in terms of total number of correct trials in the forward sequence of the visual-spatial memory span test. In summary, relative to healthy controls, AUD individuals manifested aberrant functional connectivity in the reward network, increased impulsivity, and poor neuropsychological performance in visual–spatial memory.

### 4.1. Altered Functional Connectivity across Reward Network in AUD Individuals

Addiction to drugs and alcohol involves a cascade of neuroadaptive processes, causing changes in the brain circuitries at different stages of addiction [18,89,90]. Our findings on resting state FC in the reward network indicate that AUD subjects manifested alterations in connectivity patterns, in terms of hypoconnectivity in nine connections and hyperconnectivity in three connections, involving 17 key reward structures [see Figure 7]. In particular, out of the nine reward network functional connections that showed hypoconnectivity, three were cortico–cortical connections (R.Ins–R.ACC, R.ACC–R.OFC, and R.ACC–R.PCC) in the right hemisphere, and five were subcortical–subcortical connections (R.Amg–L.Hip, L.Cdt–R.Pal, L.Tha–R.Tha, L.Cdt–L.Tha, and L.Tha–R.Ptm) involving both hemispheres, and a single inter-hemispheric subcortical-cortical connection (R.VTA–L.ACC). The three subnetworks that were hyperconnected in AUD individuals were subcortical–cortical connections, involving a left-hemispheric connection (L.NAc–L.PCC), a right-hemispheric connection (R.Pal–R.PCC), and an inter-hemispheric connection (R.Hip–L.DLP). These findings of altered brain connectivity in AUD individuals may be suggestive of neuroadaptation in the hub regions of the reward network, caused by chronic alcohol intake. In general, previous fMRI studies have reported such aberrations in resting state connectivity underlying multiple brain networks in individuals with SUD [19,28,91], including those with AUD [30,31,32,33,63,92,93,94]. Our previous study on the same sample of participants as in the current study reported that AUD individuals manifested altered default mode network (DMN) connectivity compared to controls [63]. It is clear from the findings of our past and current rsFC studies that AUD individuals manifest brain connectivity changes across neural structures involved in spontaneous, self-referential thoughts, as elicited by the DMN [95], as well as the commonly reported reward processing deficits, as elicited by the RN [10]. It is also remarkable to note that both studies showed hypoconnectivity between the ACC and PCC nodes in AUD subjects, confirming the notion of abnormal self-referential processing in these individuals [92]. Furthermore, hypoconnectivity across reward structures during resting state, as found in the current study, may also indicate a vulnerability to relapse in individuals with a history of SUD [96]. Taken together, these findings lend support to the findings of the current study, that individuals with a long history of alcohol use continue to manifest network abnormalities across the reward regions, in addition to the previously reported aberrations in DMN, even after prolonged abstinence from alcohol consumption.

The predominant pattern observed in AUD individuals was hypoconnectivity (9 out of 12 connections) across subcortical reward regions (5 connections) followed by cortical (3 connections) and cortical–subcortical (1 connection) subnetworks. Specifically, these hypoconnected nodes include key reward regions such as the VTA, amygdala, hippocampus, thalamus, pallidum, putamen, insula, ACC, PCC, and OFC, possibly indicating lower or less efficient neural communication across these subnetworks [6]. Since addiction has been characterized as a reward deficiency syndrome [97], hypoconnectivity across these reward structures in abstinent AUD subjects may indicate reduced responsiveness to rewarding stimuli during resting state, possibly due to decreased dopamine function in these individuals [50,98]. Although elevated levels of dopamine in the dorsal striatum are associated with motivation to seek and consume alcohol and drugs, long-term substance use is associated with decreased dopaminergic function, as evidenced by reductions in D2 dopamine receptors and dopamine release in the striatum in addicted subjects [50], which can also lead to reduced activity in other cortical reward regions such as the orbitofrontal cortex and cingulate gyrus, resulting in loss of control and compulsive substance use. It may be worth noting that the majority of the hypoconnected regions in AUD (9 out of 13 regions) involved the right hemisphere, implicating hemispheric asymmetry in connectivity in AUD and, hence, their functional attributes [99]. For instance, laterality studies on motivation and emotions reported that the right hemisphere responds more to unpredicted, urgent, and novel environmental events, while the left hemisphere engages with routine and habitual behaviors [100]. Therefore, it is possible that the alterations in resting state brain connectivity across reward network seen in AUD individuals have more impact on right hemispheric function, including novelty seeking and impulsivity [101].

The other FC finding was that AUD individuals manifested hyperconnectivity in three connections across five brain regions, i.e., nucleus accumbens, pallidum, hippocampus, posterior cingulate, and dorsolateral prefrontal cortex, possibly suggesting excessive and/or less focused communication during resting state among these structures. While each of these key regions is associated with distinct and shared neurocognitive processes, hyperconnectivity across these nodes during resting state can be generally interpreted as excessive rumination about reward and preoccupation with reward-related imagery or inherent reward-seeking tendencies, such as craving. Similar to our finding, a higher FC between nucleus accumbens and posterior cingulate gyrus was observed in relapsers compared to abstainers of stimulant use [96]. In addition, akin to our finding, higher hippocampal–prefrontal connectivity has also been observed in internet gaming addiction [102]. Furthermore, recent evidence implicating the pallidum as an important structure in mesocorticolimbic reward processing [103], and, therefore, the increased connectivity between pallidum and PCC, may indicate reward related tendencies (e.g., drug seeking) in the resting state. However, despite the growing number of fMRI brain connectivity studies on AUD and other SUDs during resting state and task conditions [19,28,63,94,102,104], more studies are needed to understand the exact role of specific connectivity patterns across the reward network.

### 4.2. Heightened Impulsivity in AUD Individuals

The findings of the current study also showed that motor and non-planning impulsivity components were the topmost features contributing to the classification of AUD individuals from controls. This finding reinforces the long-held notion that AUD and other externalizing traits are part of the externalizing spectrum disorders [105,106,107,108,109]. It is also known that AUD is associated with making impulsive choices during decision-making [16]. It is possible that the increased impulsivity manifested by AUD individuals may be due to altered FC across the frontal nodes [63] and/or relatively lower brain volumes of the frontal regions, as reported in our previous study [54]. Evidence from the imaging literature strongly suggest that both structural and functional aspects of the frontal lobes contribute to increased impulsivity in AUD patients [110,111,112,113]. Furthermore, recent studies have also reported associations between impulsivity and resting state measures of EEG power [114], EEG-based FC [115], and fMRI-based FC [36], suggesting that specific brain networks may mediate aspects of impulsivity in AUD, as well as other externalizing disorders. Therefore, identifying and quantifying behavioral impulsivity may contribute to improving prevention and intervention programs related to alcohol and other substance use problems [116,117]. Although attentional impulsivity was not found to significantly contribute to the classification, our previous studies [63,85] found contributions from all three components of impulsivity, while motor and non-planning aspects were top of the key features of classification, suggesting their relative importance in AUD pathology.

### 4.3. Poorer Memory Span in AUD Individuals

In the current study, the neuropsychological score “TotCor_Fw” (i.e., total number of correctly performed forward trials) from the visual span test was also identified as one of the key variables contributing to the classification of AUD individuals from controls by the RF model. On the other hand, parametric group comparison of neuropsychological variables (Table 5) revealed that the AUD group performed poorly in both “TotCor_Fw” and “Span_Fw” (i.e., span of recalled items during forward trials), compared to controls. Interestingly, these two variables tapping short-term memory performance were also found to be significant in our previous classification studies with the same sample of participants [63,85]. Furthermore, there is a strong literature support for memory deficits in individuals with chronic AUD [118,119,120,121], and some of the deficits linger even after prolonged abstinence [37]. It is also worth noting that in our previous structural MRI study on the same groups of subjects, we found that the AUD group showed lower volumes in prefrontal cortex and left hippocampus, which were associated with poorer visuospatial memory performance [54]. Furthermore, in another study on the same sample, we found that AUD subjects manifested hyperconnectivity across the parahippocampal hub regions [63], adding support to the current finding related to memory deficits. On the other hand, it is surprising that none of the scores related to executive functioning in the TOLT were significantly different from controls, possibly suggesting a partial or complete recovery of these functions in the AUD group due to abstinence. It is also possible that the current study failed to capture deficits in additional domains, as the data involved in the current study were limited to only two tests and the sample size was only modest. Future studies may employ a comprehensive and sensitive battery of neuropsychological tests in a larger sample of abstinent AUD individuals, to map neuropsychological performance in multiple domains.

### 4.4. Correlations of Significant Variables among Themselves and with Age

Correlations among the significant variables that were identified by the RF classification model revealed three highly significant positive associations among the FC features (L.Cdt–R.Pal with L.Cdt–L.Tha; L.Tha–R.Tha with L.Tha–R.Ptm; and R.Ins–R.ACC with R.ACC–R.PCC), and each of these significant pairs had a common node, i.e., L.Cdt, L.Tha, and R.ACC, respectively. While it is expected that the pairs with a common node would correlate with each other, they are also known to be structurally connected. For instance, while the caudate nucleus connects the pallidum with radial fibers [122], these basal ganglia structures have reciprocal connections with the thalamus and cortical regions and, thus, mediate cognitive and motor functions [123]. Similarly, while the ACC has both structural and functional connectivity with the PCC as part of the DMN [124], reciprocal interaction between the ACC and anterior insula serves as a major connection with the salience and reward network [125]. Interestingly, the negative correlation between memory performance (working memory) and impulsivity observed in our study was also previously reported by other studies on individuals with substance use disorders [126,127]. Lastly, as expected, non-planning and motor impulsivity were positively correlated with each other [128], and both of these dimensions were shown to be associated with craving and relapse among alcohol-dependent males [129]. On the other hand, none of the correlations between the top predictor variables and age sustained a statistical significance after multiple testing corrections, suggesting that age did not impact the classification of groups based on these top predictors. However, it is suggested that future studies may confirm these preliminary findings using a large sample of subjects involving both males and females matched on multiple characteristics, such as education, ethnicity, premorbid IQ, etc.

### 4.5. Limitations of the Current Study

The current study has several limitations. (i) The sample consisted of only males, and the findings may not be generalizable to females. In general, findings from prediction-based studies will be more useful when they are generalizable to different strata of the population from which the sample is drawn. Therefore, we suggest that future predictive studies aim for samples from both genders. (ii) The outcome groups (AUD and CTL) were not matched for age, as the age difference was statistically significant. Although age was not significantly associated with the key features, either in each group or in the total sample, the results would have been more credible if the groups were matched for age. Therefore, future studies should also aim to apply predictive models on age-matched groups, to avoid confounding the results. (iii) Finally, the sample size for a predictive model was rather small, although the random forest algorithm is known to handle such situations more effectively than other machine learning models. Therefore, similar studies with larger sample sizes are needed to confirm the findings of the present study. A larger sample is also warranted to explore associations among the features from multiple domains, since the current study did not identify potential associations among the features, possibly due to a lack of statistical power. Given these limitations, it is to be noted that the obtained results are only preliminary, while the findings of the present study might help to design future studies avoiding or mitigating these limitations.

## 5. Summary and Conclusions

The findings of the current study suggest that multidomain features drawn from the measures of brain connectivity, impulsivity, and neuropsychological tests can be successfully used in a machine learning framework to classify AUD individuals from healthy controls. In summary, our study revealed that the abstinent individuals with past AUD showed impaired brain connectivity across specific reward regions and also manifested relatively increased impulsivity and poor memory function. Evidence from the literature suggests that these anomalies may have been caused by neuroadaptation due to chronic drinking. Since treatment interventions intended to reverse these neuroadaptations show promise as potential therapeutic approaches for addiction [1], the findings of the current study may have important clinical implications. We suggest that future studies should further characterize these neural and behavioral abnormalities at multiple levels and groups, such as gender, race/ethnicity, educational attainment, socio-economic status, genomic liability, and drinking patterns, so that the findings may contribute towards personalized medicine.

## Figures and Tables

**Table 1:** Demographic and clinical characteristics of the sample.

Variable	AUD			CTL		
	N *	Mean	SD	N *	Mean	SD
Age (in years)	30	41.42	7.31	30	27.44	4.74
Education (in Years)	30	11.93	2.35	30	15.77	1.87
Age of onset (regular alcohol use)	30	15.77	2.58	12	20.50	3.80
Alcohol: Drinks/day (heavy alcohol use period)	30	12.08	10.02	12	2.88	1.93
Alcohol: Days/month (heavy alcohol use period)	30	20.30	9.01	12	3.35	3.64
Alcohol: Drinks (last 6 months)	30	2.68	6.61	18	2.61	1.98
Alcohol: Days (last 6 months)	30	3.97	8.02	18	2.94	3.62
Length of Abstinence (in months)	30	22.43	28.16	18	1.9	4.99
Tobacco: Times/day (last 6 months)	20	9.90	5.80	6	2.33	1.63
Tobacco: Days/month (last 6 months)	20	28.35	4.83	6	14.17	13.82
Marijuana: Times in last 6 months	10	98.80	91.38	4	18.75	27.61

* N refers to the number of subjects included in these mean and SD calculations for each variable. Individuals who did not consume alcohol or drugs were not included in the respective calculations.

**Table 2:** Brain regions of interest (ROI) analyzed for reward network functional connectivity.

ROI	Brain Region	Notation	Location	MNI Coordinates		
				X	Y	Z
1	L. Ventral Tegmental Area	L.VTA	Subcortical	−4	−16	−14
2	R. Ventral Tegmental Area	R.VTA	Subcortical	4	−16	−14
3	L. Nucleus Accumbens	L.NAc	Subcortical	−8	10	−10
4	R. Nucleus Accumbens	R.NAc	Subcortical	8	10	−10
5	L. Amygdala	L.Amg	Subcortical	−24	−2	−14
6	R. Amygdala	R.Amg	Subcortical	24	−2	−14
7	L. Hippocampus	L.Hip	Subcortical	−28	−10	−22
8	R. Hippocampus	R.Hip	Subcortical	28	−10	−22
9	L. Caudate	L.Cdt	Subcortical	−13	15	9
10	R. Caudate	R.Cdt	Subcortical	13	15	9
11	L. Pallidum	L.Pal	Subcortical	−18	−2	−4
12	R. Pallidum	R.Pal	Subcortical	18	−2	−4
13	L. Thalamus	L.Tha	Subcortical	−8	−12	6
14	R. Thalamus	R.Tha	Subcortical	8	−12	6
15	L. Insula (anterior)	L.Ins	Subcortical	−30	17	−15
16	R. Insula (anterior)	R.Ins	Subcortical	30	17	−15
17	L. Parahippocampal Gyrus	L.PHG	Cortical	−24	−39	−12
18	R. Parahippocampal Gyrus	R.PHG	Cortical	27	−39	−12
19	L. Anterior Cingulate Cortex	L.ACC	Cortical	−6	44	10
20	R. Anterior Cingulate Cortex	R.ACC	Cortical	6	44	10
21	L. Mid-Cingulate Cortex	L.MCC	Cortical	−6	2	40
22	R. Mid-Cingulate Cortex	R.MCC	Cortical	6	2	40
23	L. Posterior Cingulate Cortex	L.PCC	Cortical	−6	−46	30
24	R. Posterior Cingulate Cortex	R.PCC	Cortical	6	−46	30
25	L. Orbitofrontal Cortex	L.OFC	Cortical	−32	42	−16
26	R. Orbitofrontal Cortex	R.OFC	Cortical	32	42	−16
27	L. Angular Gyrus	L.Ang	Cortical	−45	−58	30
28	R. Angular Gyrus	L.Ang	Cortical	45	−58	30
29	L. Superior Parietal Lobule	L.SPL	Cortical	−24	−68	56
30	R. Superior Parietal Lobule	R.SPL	Cortical	24	−68	56
31	L. Dorsolateral PFC	L.DLP	Cortical	−44	38	19
32	R. Dorsolateral PFC	R.DLP	Cortical	44	38	19
33	L. Putamen	L.Ptm	Subcortical	−27	5	−6
34	R. Putamen	R.Ptm	Subcortical	27	5	−6

**Table 3:** Random forest importance parameters mean minimal depth, number of nodes, accuracy decrease, Gini decrease, number of trees, times a root, and *p*-value), and the direction of significance for the top significant variables (*p* < 0.05) are shown. Two of the impulsivity scores (motor and non-planning) and 12 rsFC connections were identified as important features to classify individuals into either AUD or CTL group. The variables are sorted based on the *p*-values.

Feature	Mean Minimum Depth	No. of Nodes	Accuracy Decrease	Gini Decrease	No. of Trees	Time a Root	*p*-Value	Direction
**BIS Motor Impulsivity**	1.3824	348	0.0170	2.0202	319	94	1.87E-19	A > C
**FC_3_23 (L.NAc–L.PCC)**	1.9354	330	0.0130	1.6903	295	74	1.82E-15	A > C
**FC_16_20 (R.Ins–R.ACC)**	2.0062	326	0.0149	1.5132	291	57	1.23E-14	C > A
**BIS Non-planning**	1.7619	319	0.0187	1.6830	294	74	3.05E-13	A > C
**FC_2_19 (R.VTA–L.ACC)**	2.0561	313	0.0040	1.4706	274	58	4.23E-12	C > A
**FC_20_26 (R.ACC–R.OFC)**	2.2513	299	0.0101	1.3203	272	45	1.24E-09	C > A
**FC_6_7 (R.Amg–L.Hip)**	2.3798	275	0.0063	1.1669	258	41	4.40E-06	C > A
**FC_9_12 (L.Cdt–R.Pal)**	2.5018	268	0.0039	0.9725	255	29	3.26E-05	C > A
**FC_20_24 (R.ACC–R.PCC)**	2.4732	266	0.0054	1.0002	249	28	5.58E-05	C > A
**FC_13_14 (L.Tha–R.Tha)**	2.5681	252	0.0023	0.9843	233	40	0.0016	C > A
**FC_12_24 (R.Pal–R.PCC)**	2.8735	249	0.0022	0.8844	223	20	0.0030	A > C
**FC_9_13 (L.Cdt–L.Tha)**	2.7035	249	0.0056	0.9541	228	34	0.0030	C > A
**FC_13_34 (L.Tha–R.Ptm)**	2.7980	247	0.0053	0.9212	234	24	0.0044	C > A
**FC_8_31 (R.Hip–L.DLP)**	2.9451	238	0.0047	0.8020	217	20	0.0220	A > C

*Acronym:* BIS—Barratt Impulsiveness Scale, FC—Functional Connectivity, R—Right, L—Left, ACC—Anterior Cingulate Cortex, Amg—Amygdala, Ang—Angular Gyrus, Cdt—Caudate, DLP—Dorsolateral PFC, Hip—Hippocampus, Ins—Insula (anterior), NAc—Nucleus Accumbens, OFC—Orbitofrontal Cortex, Pal—Pallidum, PCC—Posterior Cingulate Cortex, PHG—Parahippocampal Gyrus, Ptm—Putamen, Tha—Thalamus, VTA—Ventral Tegmental Area. Key: A > C = AUD > CTL; C > A = CTL > AUD.

**Table 4:** Pearson bivariate correlations between the age of the participant and the top significant variables of the RF model. Correlation coefficient (r) and *p*-values (before Bonferroni correction) are provided for alcohol use disorder (AUD), control (CTL) group, and the total sample (ALL). None of the variables survived Bonferroni correction for multiple comparisons. Zero-order correlations were used for each group separately (N = 30) and partial correlations controlling for group effects were used for the all sample (N = 60).

Feature	AUD (N = 30)		CTL (N = 30)		^§^ ALL (N = 60)	
	r	*p*	r	*p*	r	*p*
FC_2_19 (R.VTA–L.ACC)	−0.08	0.6744	0.22	0.2449	0.03	0.8131
FC_3_23 (L.NAc–L.PCC)	0.16	0.3949	−0.21	0.2693	0.02	0.8956
FC_6_7 (R.Amg–L.Hip)	−0.02	0.8993	0.38	0.0374 *^○^	0.11	0.4225
FC_8_31 (R.Hip–L.DLP)	0.16	0.3977	0.27	0.1489	0.20	0.1276
FC_9_12 (L.Cdt–R.Pal)	0.16	0.3964	−0.01	0.9583	0.09	0.5079
FC_9_13 (L.Cdt–L.Tha)	−0.15	0.4430	0.05	0.7812	−0.06	0.6349
FC_12_24 (R.Pal–R.PCC)	0.01	0.9579	−0.09	0.6402	−0.03	0.8298
FC_13_14 (L.Tha–R.Tha)	−0.26	0.1616	−0.02	0.9168	−0.15	0.2588
FC_13_34 (L.Tha–R.Ptm)	0.07	0.7127	0.12	0.5196	0.09	0.5002
FC_16_20 (R.Ins–R.ACC)	−0.19	0.3149	0.09	0.6203	−0.07	0.6062
FC_20_24 (R.ACC–R.PCC)	−0.05	0.7849	0.01	0.9574	−0.03	0.8195
FC_20_26 (R.ACC–R.OFC)	0.19	0.3182	0.11	0.5460	0.16	0.2110
BIS_NP (Non-planning)	0.03	0.8936	0.21	0.2644	0.09	0.4815
BIS_MI (Motor Impulsivity)	0.23	0.2121	0.12	0.5432	0.20	0.1268

* *p* < 0.05 (before Bonferroni correction); ^○^ Not significant after Bonferroni correction; ^§^ Based on partial correlation adjusted for group effect. Refer to Table 2 for the details of the ROIs in the FC variable.

**Table 5:** Comparison of neuropsychological variables between AUD or CTL group using one-way ANOVA.

	AUD		CTL		F	*p*
	Mean	SD	Mean	SD		
ExcMovMade_All	15.04	17.02	7.83	6.66	4.43	0.0402 *
AvgPicTime_All	3.09	1.09	2.81	0.96	1.02	0.3167
AvgTotTime_All	5.16	1.58	4.72	1.64	1.01	0.3199
TotTrlTime_All	482.60	178.18	404.24	139.05	3.29	0.0755
AvgTrlTime_All	22.98	8.48	19.25	6.62	3.29	0.0755
TotCor_Fw	7.00	2.58	10.21	2.78	19.06	**0.0001 ++**
TotCor_Bw	6.31	3.02	8.31	1.87	8.95	0.0042 *
Span_Fw	5.44	1.33	6.83	1.36	14.25	**0.0004 ++**
Span_Bw	4.65	1.44	5.52	0.95	7.02	0.0106 *
TotAvgTime_Fw	26.72	9.13	28.31	10.53	0.35	0.5591
TotAvgTime_Bw	17.72	9.39	17.79	10.01	0.00	0.9791
TotCorAvgTime_Fw	38.15	12.39	32.48	8.07	4.06	0.0490 *
TotCorAvgTime_Bw	28.93	13.92	27.16	10.66	0.28	0.5963

* Significant before Bonferroni correction; ++ Significant after Bonferroni correction.

## Data Availability

The data presented in this study are available in Supplementary Material.

## Supplementary Materials

The following supporting information can be downloaded at: https://www.mdpi.com/article/10.3390/bs12050128/s1.

## Appendix A

**Table A1:** List of features (F) included in the random forest classification model: (i) 21 reward network connections identified by the feature selection process (F1–F21), 13 neuropsychological scores involving 5 TOLT scores (F22–F26) and 8 VST scores (F27–F34), and 3 BIS scores (F35–F37).

F#	Feature	Detail
1.	FC_2_19 (R.VTA–L.ACC)	FC between R. Ventral Tegmental Area and L. Anterior Cingulate Cortex
2.	FC_3_23 (L.NAc–L.PCC)	FC between L. Nucleus Accumbens and L. Posterior Cingulate Cortex
3.	FC_6_11 (R.Amg–L.Pal)	FC between R. Amygdala and L. Pallidum
4.	FC_6_7 (R.Amg–L.Hip)	FC between R. Amygdala and L. Hippocampus
5.	FC_8_31 (R.Hip–L.DLP)	FC between R. Hippocampus and L. Dorsolateral Prefrontal Cortex
6.	FC_9_12 (L.Cdt–R.Pal)	FC between L. Caudate and R. Pallidum
7.	FC_9_13 (L.Cdt–L.Tha)	FC between L. Caudate and L. Thalamus
8.	FC_9_18 (L.Cdt–R.PHG)	FC between L. Caudate and R. Parahippocampal Gyrus
9.	FC_9_23 (L.Cdt–L.PCC)	FC between L. Caudate and L. Posterior Cingulate Cortex
10.	FC_9_27 (L.Cdt–L.Ang)	FC between L. Caudate and L. Angular Gyrus
11.	FC_12_24 (R.Pal–R.PCC)	FC between R. Pallidum and R. Posterior Cingulate Cortex
12.	FC_13_14 (L.Tha–R.Tha)	FC between L. Thalamus and R. Thalamus
13.	FC_13_34 (L.Tha–R.Ptm)	FC between L. Thalamus and R. Putamen
14.	FC_16_20 (R.Ins–R.ACC)	FC between R. Insula and R. Anterior Cingulate Cortex
15.	FC_17_31 (L.PHG–L.DLP)	FC between L. Parahippocampal Gyrus and L. Dorsolateral Prefrontal Cortex
16.	FC_20_24 (R.ACC–R.PCC)	FC between R. Anterior Cingulate Cortex and R. Posterior Cingulate Cortex
17.	FC_20_26 (R.ACC–R.OFC)	FC between R. Anterior Cingulate Cortex and R. Orbitofrontal Cortex
18.	FC_20_31 (R.ACC–L.DLP)	FC between R. Anterior Cingulate Cortex and L. Dorsolateral Prefrontal Cortex
19.	FC_21_24 (L.MCC–R.PCC)	FC between L. Middle Cingulate Cortex and R. Posterior Cingulate Cortex
20.	FC_22_33 (R.MCC–L.Ptm)	FC between R. Middle Cingulate Cortex and L. Putamen
21.	FC_28_29 (L.Ang–L.SPL)	FC between L. Angular Gyrus and L. Superior Parietal Lobule
22.	ExcMovMade_All	Overall excess moves beyond the minimum moves required to solve the puzzle
23.	AvgPicTime_All	Overall average pickup time to solve the puzzle
24.	AvgTotTime_All	Overall average total time to solve the puzzle
25.	TotTrlTime_All	Overall total trial time within each puzzle type
26.	AvgTrlTime_All	Overall average trial time across trials per puzzle type
27.	TotCor_Fw	Total number of correctly performed trials in forward sequence
28.	TotCor_Bw	Total number of correctly performed trials in backward sequence
29.	Span_Fw	Maximum span or sequence-length achieved in forward sequence
30.	Span_Bw	Maximum span or sequence-length achieved in backward sequence
31.	TotAvgTime_Fw	Total average time taken across all trials performed in forward sequence
32.	TotAvgTime_Bw	Total average time taken across all trials performed in backward sequence
33.	TotCorAvgTime_Fw	Total correct average time taken across all correct trials in forward sequence
34.	TotCorAvgTime_Bw	Total correct average time taken across all correct trials in backward sequence
35.	BIS_AI	Barratt Impulsiveness Scale, Attentional Impulsivity Score
36.	BIS_MI	Barratt Impulsiveness Scale, Motor Impulsivity Score
37.	BIS_NP	Barratt Impulsiveness Scale, Non-planning Impulsivity Score
